# Mutations affecting interaction of integrase with TNPO3 do not prevent HIV-1 cDNA nuclear import

**DOI:** 10.1186/1742-4690-8-104

**Published:** 2011-12-16

**Authors:** Alexandra Cribier, Emmanuel Ségéral, Olivier Delelis, Vincent Parissi, Aurélie Simon, Marc Ruff, Richard Benarous, Stéphane Emiliani

**Affiliations:** 1 Inserm, U1016, Institut Cochin, Paris, France; 2 CNRS, UMR 8104, Paris, France; 3 Université Paris Descartes, Paris, France; 4 LBPA, CNRS, ENS de Cachan, Cachan, France; 5 Laboratoire MFP, UMR 5234-CNRS, Université de Bordeaux Victor Segalen, Bordeaux, France; 6 IGBMC (Institut de Génétique et de Biologie Moléculaire et Cellulaire), Département de Biologie et de Génomique Structurales, UDS, CNRS, INSERM, Illkirch, France; 7 Mutabilis (S.A.), Romainville, France

## Abstract

**Background:**

Integration of human immunodeficiency virus type 1 (HIV-1) into a host cell chromosome is an essential step under the control of the viral integrase (IN). Although this enzyme is necessary and sufficient to catalyze the integration reaction *in vitro*, cellular cofactors are involved in the process *in vivo*. The chromatin-associated factor LEDGF/p75 interacts with IN and promotes integration to transcription units of the host genome. HIV-1 IN also binds the karyopherin TNPO3, however the significance of this interaction during viral replication remains to be explored.

**Results:**

Here we present a functional analysis of IN mutants impaired for LEDGF/p75 and TNPO3 interaction. Among them, IN W131A and IN Q168L, that were previously identified to be deficient for LEDGF/p75 interaction, were also partially impaired for TNPO3 binding. We observed that mutations abolishing IN ability to form tetramers resulted in a severe reduction in LEDGF/p75 binding. In sharp contrast, no correlation could be found between the ability of IN to multimerize and TNPO3 interaction. Most of the mutant viruses were essentially impaired for the integration step whereas the amount of 2-LTR circles, reflecting the nuclear import of the viral DNA, was not significantly affected.

**Conclusion:**

Our functional analysis of HIV-1 IN mutants reveals distinct structural basis for TNPO3 interaction and suggests that the interaction between IN and TNPO3 is not a major determinant of nuclear import but could take place at a nuclear step prior to integration.

## Background

Following the entry of the HIV-1 viral core into the cytoplasm of a target cell, reverse transcription of the retroviral RNA into a linear double strand cDNA copy takes place within the reverse transcription complex (RTC) [1,2]. Once synthesized by the RTC, this viral cDNA becomes part of a large nucleoprotein complex called the pre-integration complex (PIC) [3-5]. HIV-1 PICs retain components of the RTC: nucleocapsid (NC), integrase (IN), matrix (MA), Vpr and reverse transcriptase (RT) [3,6], together with cellular cofactors. This complex is transported to the nucleus where the viral cDNA integrates into a host cell chromosome, a key step that is under the control of the retroviral enzyme IN [7-9]. Whereas the recombinant HIV-1 IN protein is sufficient to catalyze the 3' processing and strand transfer activities for *in vitro *integration, functional interactions between IN and host cell factors are required during the early events of HIV-1 replication *in vivo *[10-12].

HIV-1 host cell factor dependency is well illustrated by LEDGF/p75, a cellular chromatin-associated protein involved in transcriptional regulation of cellular genes that were identified as an IN interacting factor [11,13,14]. LEDGF/p75 plays an important role in lentiviral cDNA integration, as demonstrated by mutagenesis [14-17], over-expression of LEDGF/p75 IBD (Integrase Binding Domain) [18,19], as well as RNAi and knock-out studies [18-23]. Structural studies revealed the roles of both the catalytic core domain (CCD) dimeric interface as well as the N-terminal domain (NTD) of IN for high affinity binding to IBD [16,24,25]. In addition, LEDGF/p75 stimulates the formation of IN tetramers, which are required for efficient concerted integration of both viral ends into host DNA [26-29]. Furthermore, the targeting of viral integration to specific regions of the host chromosome is under the control of LEDGF/p75. Indeed, LEDGF/p75 guides the site selection of lentiviral integration within transcription units (TUs) of transcriptionally active genes, while GpC islands and promoter regions are disfavored [20,21,30]. Albeit not strictly essential for replication, LEDGF/p75 tethers PIC-associated IN to chromatin to presumably stimulate its enzymatic activity at the site of integration [31,32].

In contrast to the gammaretrovirus Moloney murine Leukemia Virus (MLV), HIV-1 and other lentiviruses possess the ability to infect both dividing and non-dividing cells [33,34]. This specificity is particularly important to explain the infection of terminally differentiated cells, including macrophages and microglia that constitute an important reservoir of virus in infected individuals [35]. In these cells, the mechanisms leading to the establishment of an integrated provirus are still unclear. One key step could be the nuclear import of the PIC, which must be translocated through the intact nuclear membrane in an energy dependent manner. To pass through the nuclear pore complex (NPC), components of the PIC must interact with cellular nucleo-cytoplasmic transport factors as well as NPC components such as nucleoporins (NUPs). Several viral determinants, including karyophilic signals of MA, Vpr or IN, as well as the viral flap DNA structure could contribute to the nuclear import of the PIC; however, their exact role is still debated (see [36-38] for recent reviews). In contrast, CA recently emerged to be a key factor in conferring the ability of HIV-1 to infect non-dividing cells. While evidence of a direct interaction between CA and the nuclear import machinery is still lacking, CA uncoating of the core appears to be coupled with an active nuclear translocation [39-41]. CA dissociation could allow subsequent interactions of viral karyophilic motifs with nuclear import factors. Alternatively, it was observed that intact capsid lattices remain associated with the PIC and that uncoating of the core occurs at the nuclear pore upon completion of the reverse transcription [42].

The cellular factors involved in PIC nuclear import have been the focus of numerous and often contradictory studies. Members of the importin α protein family were previously shown to interact with IN [43-50]. Importin 7, a nucleocytoplasmic factor related to importin β, was also implicated in this process by directly interacting with IN [48,51,52]. In addition to karyopherins, several components of the nuclear pore complex (NPC) were identified to play a role in early steps of HIV-1 replication [53-57].

Using genome-wide RNAi knock down approaches, TNPO3 was recently identified as a HIV-dependency factor involved at a stage between reverse transcription and integration [53,55]. TNPO3 belongs to the importin β family that plays the role of transporter of serine/arginine rich (SR) proteins into the nucleus [58-61]. TNPO3 was shown to mediate nuclear import of the PIC, both in dividing and non-dividing cells [62]. We identified TNPO3 as a partner of IN by yeast two-hybrid (y2H) screenings using IN as a bait, and rebound screenings using an HIV-1 library confirmed that TNPO3 interacts solely with IN [62,63]. Whereas a direct interaction between HIV-1 IN and TNPO3 was confirmed by different studies [62-64], its relevance in HIV-1 replication has been questioned. For instance, no correlation could be found between the affinity of TNPO3 for different retroviral INs and its requirement during infection [64,65]. Instead, studies pinpointed to a dominant role of CA for TNPO3 dependency [64,66].

Here, we assessed the role of TNPO3-IN interaction in HIV-1 replication. Site-directed mutagenized IN proteins were analyzed for LEDGF/p75 and TNPO3 interaction. The structure function analysis of these mutants revealed that mutations previously characterized to impair LEDGF/p75 binding also affected interaction with TNPO3. However, contrary to what was observed for LEDGF/p75, TNPO3 binds IN regardless to its tetrameric state. Importantly, while impairing HIV-1 replication, none of these mutations significantly affected amounts of 2-LTR circles, indicating a lack of correlation between the ability of IN to interact with TNPO3 and nuclear import of HIV-1.

## Results

**Figure 1:**
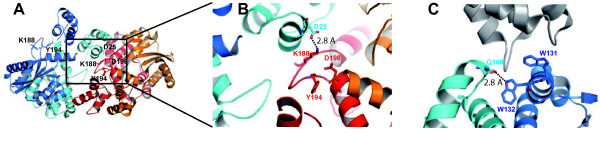
**HIV-1 integrase mutants.** (A) Crystal structure of an IN_NTD-CCD _tetramer ABCD (PDB ID 1k6y, [89]) represented in ribbons. Individual subunits of IN are colored *blue*, *cyan*, *red*, and *gold*. (B) At the NTD-CCD interface, the ε-amine of Lys188 is located at 2.8 Å from Asp25 making and H-bond and at 4.5 Å from Glu198 (also indicated). (C) IN_CCD _LEDGF/p75_IBD _interface (PDB ID 2b4j, [24]). Individual subunits of IN are colored in *blue *and *cyan*, LEDGF/p75_IBD _is colored in *grey*.

Recent retroviral intasome structures revealed a tetramer of IN formed by a dimer-of-dimers [28,67]. To characterize whether the tetramerization of HIV-1 IN was involved in TNPO3 interaction, we analyzed mutants of IN that are affected in their abilities to multimerize. Residues Lys188 and Tyr194 within the CCD of IN are located within a loop connecting CCD helices α5 and α6 involved in tetramerization [26] (Figure 1A). Lys188 forms a salt bridge with Asp25 at the intermolecular NTD-CCD interface, while Tyr194 is involved in the hydrophobic interaction at the dimer-dimer interface [26,27] (Figure 1B). Alongside these residues, we also included in our assays two previously characterized IN mutants W131A and Q168L that were deficient for LEDGF/p75 binding [14,15,17]. The residue Q168 does not interact directly with LEDGF/p75, but hydrogen-bond with W132 from the opposite IN monomer, therefore participating in the dimerization of IN (Figure 1C). The residue W131 is directly involved in hydrophobic contacts with LEDGF/p75 residues F406 and V408 [16,24-26].

**Figure 2:**
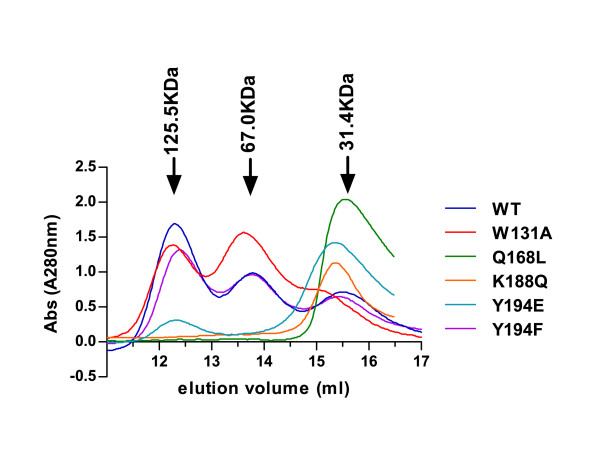
**Oligomerization properties of WT and mutant INs**. Size exclusion chromatography (SEC) elution profiles of IN proteins (2.5 μM) versus elution volumes of protein standards (black arrows) are represented. The SEC profile of WT IN (dark blue) shows three peaks corresponding to tetramers, dimers and monomers, respectively. SEC profiles of IN mutants W131A, Q168L, K188Q, Y194E and Y194F are shown in red, green, orange, cyan and purple, respectively.

Purified HIV-1 IN exists in solution as a mixture of multiple oligomerized forms containing monomers, dimers and tetramers, even in absence of DNA. We used size exclusion chromatography (SEC) to assess the multiple multimeric states of our tag-free IN mutants. In this assay, wild type IN was predominantly tetrameric, with the presence of two smaller peaks corresponding to the dimeric and monomeric forms. IN W131A behaved mostly as a mixture of tetramers and dimers. In sharp contrast, mutation Q168L disrupting the Q168-W132 H-bond results in a major shift to a low molecular weight peak corresponding exclusively to monomers. Substitution of the Lys188 residue to a Gln, targeting the Asp25:Lys188 intra-molecular salt bridge, also led to a monomeric form of IN. A recent crystal structure of IN tetramers pinpointed a role of residue Tyr194 in the closure of the dimer-dimer interface [26]. Substitution of Tyr194 by hydrophobic residue Phe resulted in an elution profile similar to wild type IN. Remarkably, mutation Y194E led to a pronounced shift to monomeric forms. These results are in good agreement with previous reports highlighting a role of the 186-195 loop in the tetramerization of IN [26,27] (Figure 2).

**Figure 3:**
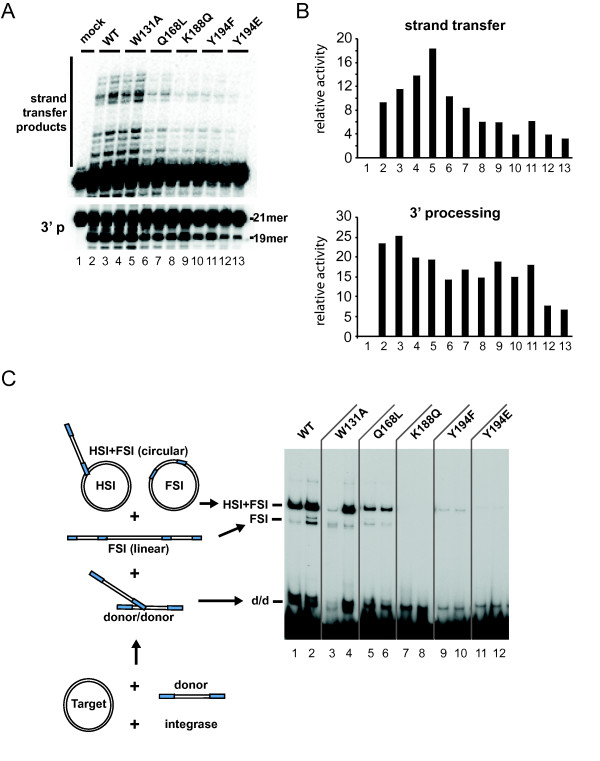
**Enzymatic activities of WT and mutant INs**. (A) 3' processing and strand transfer activities of IN mutants. Using an oligonucleotide-based assay, activities of recombinant INs were tested at two different concentrations: 100 nM (lanes 2, 4, 8, 10, 12); or 120 nM (lanes 3, 5, 7, 9, 11, 13). Reaction products were separated in a denaturing urea gel and visualized with a STORM PhosphorImager (Molecular Dynamics). (B) Quantification of 3'-processing and strand transfer activities of IN mutants. Gels from figure 3A were quantified with Image Quant™ 4.1 software. (C) Concerted integration assay was performed using IN (2 pmoles, lanes 1, 3, 5, 7, 9, 11 and 5 pmoles, lanes 2, 4, 6, 8, 10, 12), 150 ng of naked acceptor DNA (3000 bp) and 15 ng of ^32^P 5'-labelled donor pre-processed DNA (296 bp). The reaction products were loaded on 1% agarose. The positions of the different products obtained after half-site integration (HSI), full-site integration (FSI) and donor/donor integration (d/d) are reported.

HIV-1 IN concerted integration activities are closely linked to its multimerization state [68-70]; thus, we next analyzed the effects of these mutations on the reactions catalyzed *in vitro *by the retroviral enzyme. We first assessed the effect of these mutations on intrinsic 3' processing and strand transfer activities. The W131A, Q168L, K188Q and Y194F IN mutants retained near wild type 3' processing activity, while the Y194E mutant was impaired (Figure 3A lower panel and 3B lower panel). The W131A mutant also displayed wild type level of strand transfer activity. The mutations Q168L, K188Q and Y194F partially decreased strand transfer activity. Again, mutant Y194E was more severely affected (Figure 3A upper panel and 3B upper panel). We next assessed the ability of these mutants to perform *in vitro *concerted integration under previously reported conditions [68]. Using a 296 bp DNA substrate containing the 21 nt of both pre-processed HIV-1 U3 and U5 viral ends at each extremity, IN WT displayed a robust activity generating all the expected integration products including the half site integration product (HSI) and the full site integration product (FSI). IN W131A displayed a strong activity at 5 pmoles. Surprisingly, despite its monomeric elution profile in SEC (Figure 2), Q168L retained a significant catalytic activity. In sharp contrast, K188Q, Y194F and Y194E were almost incapable of concerted integration activities (Figure 3C).

**Figure 4:**
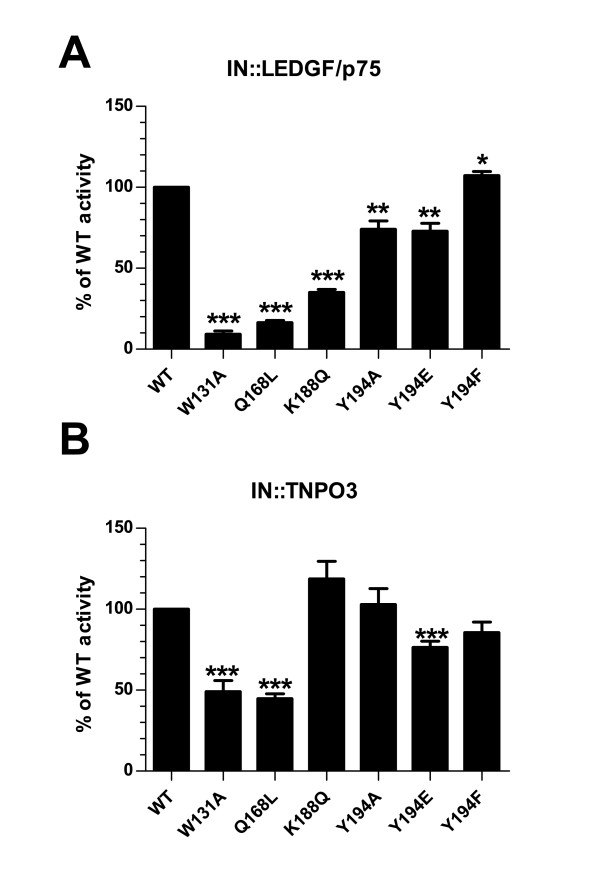
**In vitro interaction assay of IN mutants with LEDGF/p75 and TNPO3**. Interaction between INs and LEDGF/p75 (A) or TNPO3 (B) was measured by HTRF. GST-LEDGF/p75 (5 nM) or GST-TNPO3 (5 nM) and His-IN WT or indicated IN mutants (80 nM) were incubated with anti-GST-Europium Cryptate and anti-His-XL antibodies. HTRF signal was read at 2 h post incubation in a Pherastar (BMG) at 665 and 620 nm after excitation at 337 nm. HTRF signals of indicated IN mutants were expressed as a percent IN WT ΔF. Results are an average of three (IN::LEDGF/p75) or four (IN::TNPO3) experiments performed in duplicate. Statistical significance values were calculated using a student two-sided *t *test. ***, *P *< 0.0001; **, *P *< 0.001; *, *P *< 0.01.

In order to assess the impact of IN multimerization on LEDGF/p75 and TNPO3 binding, we first confirmed that our recombinant proteins were able to interact in a Ni-NTA pull-down assay. As expected, His-tagged wild type IN interacted with both full length GST-LEDGF/p75 and GST-TNPO3, while the GST protein alone failed to be pulled down. IN also recovered the IBD of LEDGF/p75 that was previously identified in yeast 2-hybrid screenings [14] (Additional file 1: Figure S1). In the absence of His-IN, none of the GST fusion proteins were recovered (data not shown). Next, we established a more quantitative assay to assess the binding properties of our mutants. *In vitro *interactions were studied by HTRF, a FRET-based assay using His-IN and either LEDGF/P75 or TNPO3 constructs tagged with GST. Recombinant proteins were incubated to form a complex in presence of anti-GST-EuCryptate/anti-His-XL665, and the transfer of energy between the two proteins yields a fluorescent signal only if they are in close proximity, *i.e*. if they are physically interacting. IN mutants were first characterized for their ability to bind LEDGF/p75. The His-IN::GST-LEDGF/p75 complex yielded a strong and reproducible signal reflecting the robustness of this assay. As previously reported, both IN W131A and IN Q168L displayed a weak interaction with LEDGF/p75 (9.3 and 16.3% of IN WT interaction in our assay, respectively). Substitution of Lys188 for a Gln also resulted to a 64.9% decrease of the binding to LEDGF/p75. While IN Y194F interacted with LEDGF/p75 at wild type level, the substitution of Tyr for a negatively charged Glu or a neutral Ala moderately diminished IN binding to LEDGF/p75 by 26 and 27% respectively (Figure 4A).

The same assay was developed to quantify IN::TNPO3 interaction. Both IN mutants K188Q and Y194A interacted with TNPO3 as well as IN WT, while the substitution of Y194 to Phe or Glu resulted in a 14.4 and 23.5% decrease of TNPO3 binding. Surprisingly, the strongest decrease for TNPO3 binding was observed for both LEDGF/p75 interaction-defective integrase IN W131A and IN Q168L (49% and 45% of IN WT, respectively) (Figure 4B).

**Figure 5:**
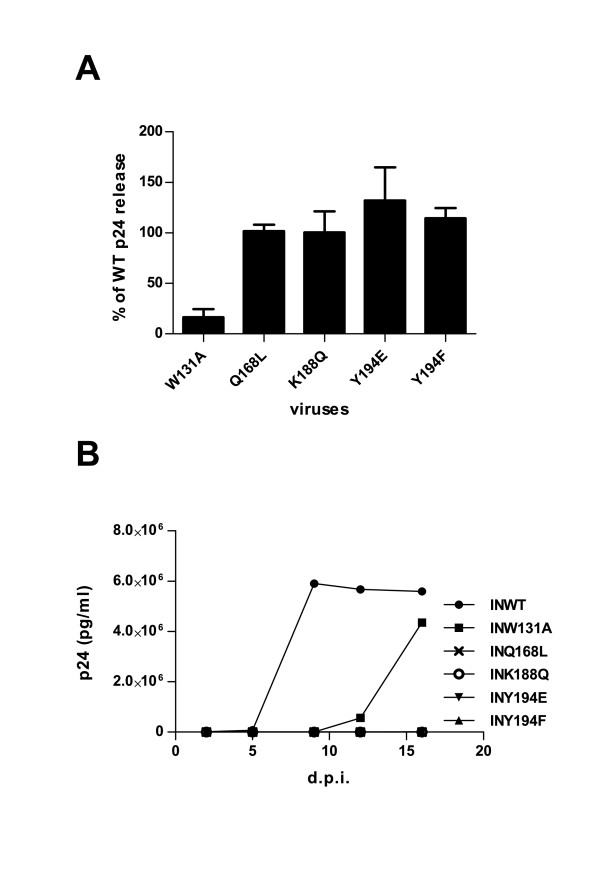
**Analysis of viral production and replication**. (A) 293T cells were transfected with the WT or mutant pBru molecular clones. Viral release was monitored by quantification of CAp24 antigen in the supernatant of the producer cells 48 h after transfection. Results are represented as a percentage of WT CAp24 release. Error bars represent standard deviations of duplicate transfections. (B) SupT1 cells were infected with equal amounts of viruses. Viral replication was followed at indicated d.p.i. by measuring CAp24 antigen in the supernatant of infected cells.

In order to determine the impact of these mutations on the viral replication in infected cells, they were introduced into the replicative Bru molecular clone; and viral stocks were produced. Among the IN mutants virus, only HIV-1 W131A displayed a severe reduction of viral particle release, yielding 16% of wild-type Cap24 release (Figure 5A). To assess the ability of IN mutant viruses to replicate, SupT1 cells were infected with equal amount of WT or mutant viruses, and viral replication was monitored by measuring p24 antigen in the supernatant of infected cells. Compared to the wild-type virus, HIV-1 W131A showed a 7 days delay in breakthrough whereas HIV-1 Q168L, HIV-1 K188Q, and HIV-1 Y194E and HIV-1 Y194F mutants were replication-deficient (Figure 5B).

**Figure 6:**
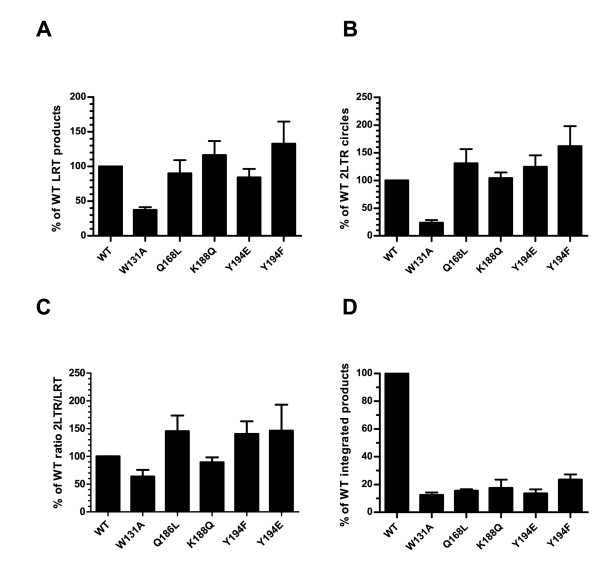
**IN mutants are primarily impaired at the integration step**. SupT1 cells were infected in a single round experiment with equal amounts of WT or mutants Bru viruses. At 7 h, 24 h and 48 h post infection (p.i.), cellular DNA was extracted and quantified for (A) late reverse transcribed (LRT) viral cDNA products at 7 h p.i., (B) 2-LTR circles at 24 h p.i., and (D) integrated proviral DNA at 48 h p.i., using a real time quantitative PCR. Results are expressed as the percentage of WT products. Depending on the mutant, error bars represent standard deviations of n = 2 to n = 6 experiments performed in duplicate. (C) 2-LTR circles were normalized to LRT viral cDNA products.

We analyzed the impact of IN mutations on early stages of HIV-1 replication using real-time quantitative PCR. Preliminary observations in HeLa cells indicated that some of our IN mutant viruses displayed strong cDNA synthesis defects when pseudotyped with the vesicular stomatitis virus glycoprotein (VSVg) (Additional file 2: Figure S2). Interestingly, these defects could be compensated when viruses entered cells by fusion using HIV-1 gp120 envelope suggesting that IN mutations could affect pre-integration steps specific to the VSVg-mediated endocytic pathway (Figure 6A and Additional file 2: Figure S2). Thus, utilizing real-time quantitative PCR, we first measured late reverse transcribed (LRT) viral cDNA products, 2-LTR circles and integrated proviral DNA in SupT1 cells infected with IN mutant viruses encoding HIV-1 *env*. In this experiment, virus replication was restricted to a single round infection by addition of Saquinavir, a protease inhibitor. At 7 h post infection, HIV-1 WT, HIV-1 Q168L, HIV-1 K188Q, HIV-1 Y194E and HIV-1 Y194F viruses synthesized similar amount of LRT viral cDNA products, whereas HIV-1 W131A displayed a 2.7-fold defect (Figure 6A). Detection of 2-LTR circle forms of viral HIV-1 cDNA is indicative of nuclear import of the viral genome. At 24h p.i., HIV-1 Q168L, HIV-1 K188Q, HIV-1 Y194E and HIV-1 Y194F mutant viruses converted equal or slightly higher amounts of 2-LTR circles (1.3-fold, 1.0-fold, 1,2-fold and 1.6-fold, respectively) compared to the wild type virus whereas HIV-1 W131A yielded an approximate 4-fold reduction (Figure 6B). When normalized to the quantity of LRT viral cDNA products , HIV-1 W131A displayed about 63% of the wild-type 2-LTR circles level, whereas HIV-1 Q168L, HIV-1 K188Q, HIV-1 Y194E and HIV-1 Y194F displayed about 145%, 90%, 140% and 146% of the HIV-1 WT, respectively (Figure 6C). Finally, levels of integrated proviruses at 48 h p.i. were severely reduced for all the mutants (12 to 24% of that of the wild type), indicating that these mutations primarily impact the integration step (Figure 6D).

**Figure 7:**
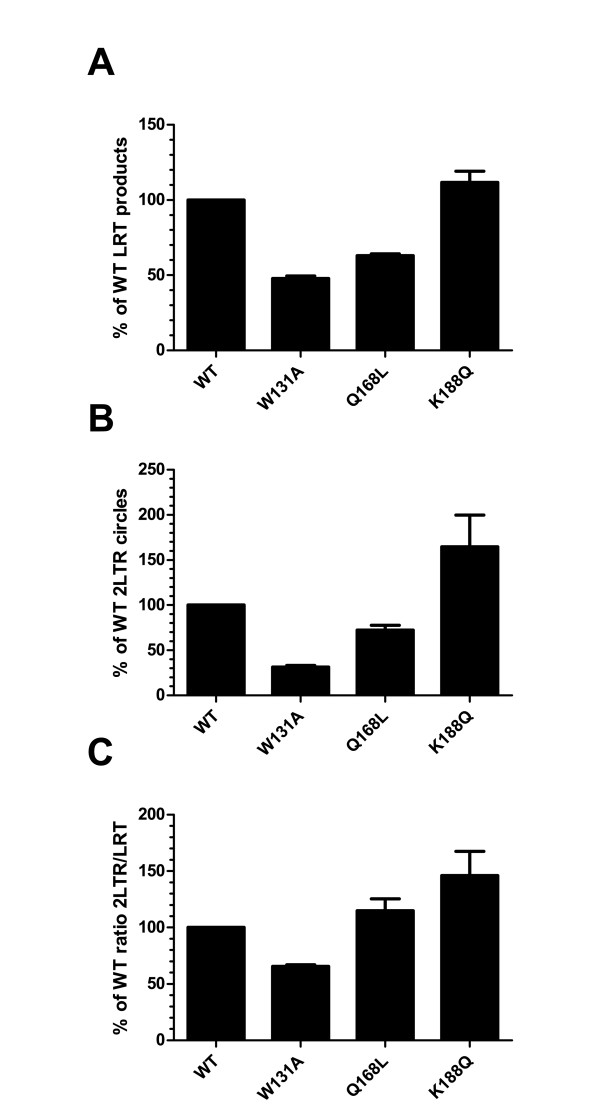
**IN mutants are not significantly affected at the nuclear import step**. SupT1 TL34 cells depleted for LEDGF/p75 were infected in a single round experiment with equal amounts of WT or mutants Bru viruses. Cellular DNA was extracted and quantified for (A) late reverse transcribed (LRT) viral cDNA products at 7 h p.i., (B) 2-LTR circles at 24 h p.i., using a real time quantitative PCR. (C) 2-LTR circles were normalized to LRT viral cDNA products. Error bars represent standard deviations of n = 2 experiments performed in duplicate. Results are expressed as the percentage of WT products.

IN mutations affecting both nuclear import and integration could result in opposite effects on the accumulation of 2-LTR circles in the nucleus. Because IN mutants W131A and Q168L are impaired for interaction with both TNPO3 and LEDGF/p75, we decided to assess more precisely the impact of these mutations on nuclear import in the absence of LEDGF/p75. Using LEDGF/p75-depleted SupT1 cells [71], we quantified 2-LTR circles levels upon infection with our mutant viruses. LRT viral cDNA products and 2-LTR circles were quantified at 7 h and 24 h p.i., respectively. For this experiment, LRT viral cDNA products were quantified using primers and a Taqman probe within the *env *region [72]. When compared to the WT virus, HIV-1 W131A and HIV-1 Q168L displayed about 50.7% and 36.9% decrease in LRT viral cDNA products, respectively. In contrast, HIV-1 K188Q synthesized amounts of reverse transcribed viral cDNA products similar to wild type level (111.6%) (Figure 7A). At 24 h p.i., HIV-1 W131A and HIV-1 Q168L displayed 31.3% and 72.4% of wild type levels of 2-LTR circles, respectively, whereas HIV-1 K188Q displayed a 1.6-fold increase (Figure 7B). When normalized to the quantity of LRT viral cDNA products, HIV-1 W131A, HIV-1 Q168L and HIV-1 K188Q converted 65.5%, 114.9% and 146.0% of their LRT viral cDNA products in to 2-LTR circles, respectively (Figure 7C). Together, these data suggest that IN mutations reducing TNPO3 binding have a rather limited effect (W131A) or no effect (Q168L) on nuclear import of the PIC.

## Discussion

Site-direct mutagenesis experiments previously identified LEDGF/p75-binding deficient IN mutants [14,15,17]. Among the residues analyzed in this study, W131 is the only one that makes a direct contact with LEDGF/p75 IBD. W131 forms hydrophobic contacts with LEDGF/p75 residues F406 and V408. The side chain of the surface exposed W131 residue was shown to rotate upon LEDGF/p75 binding [24], and mutation of this residue significantly impaired LEDGF/p75 interaction [17,73]. The elution profile of W131A resembled the one of IN WT consisting of a mixture of mono-, di- and tetramers with a predominance of dimeric forms (Figure 2). Consistent with this observation, IN W131A reached near WT concerted integration activity at its highest concentration (Figure 3B).

Our structure-function analysis of IN mutants identified residues important for the oligomerization of IN. Mutations Q168L, K188Q and Y194E that destabilized IN multimers also affected LEDGF/p75 binding in our HTRF assay. Whereas the Q168 residue is not directly binding LEDGF/p75 IBD, it forms a hydrogen bound with W132 that is important for the IN dimer interface and mutations altering Q168 impaired LEDGF/p75 binding (Figure 4A). The Q168L mutant behaved solely as a monomer, confirming that alteration of the conformation of the CCD α4/α5 connector reduces IN affinity for LEDGF/p75 (Figure 4A). The result of this conformational alteration provides an explanation for the replication defect previously described for HIV-1 Q168L ([14,17,73] and Figure 5B). The residues K188 and Y194 are within the constrained loop contributing to the IN tetrameric interface [26,27]. Consistent with previous results, we found that IN K188Q mutant was predominantly monomeric (Figure 2) and showed almost 3-fold decrease for LEDGF/p75 interaction (Figure 4A). Mutation Y194E was also compromised for tetramer formation, although to a lesser extent, as tetramers forms were still present. Accordingly, this mutation resulted in a 25% decreased affinity for LEDGF/p75. In sharp contrast, Y194F displayed WT SEC profile and retained high affinity for LEDGF/p75.

On the other hand, this mutagenesis study could not establish a correlation between the formation of IN tetramers and TNPO3 binding. Indeed, W131A and Q168L displayed the strongest decreases in TNPO3 binding while showing opposite oligomerization profiles. Furthermore, the ability of monomeric IN to interact with TNPO3 was further confirmed with the mutant K188Q. These results suggest that TNPO3 could bind to a surface of monomeric IN that remains exposed after tetramerization. Despite its SEC monomeric profile, the Q168L mutant retains some catalytic activity *in vitro*. However this mutation must alter the overall conformation of the protein and its ability to interact with cellular cofactors. On the contrary, the W131A mutant displayed *in vitro *catalytic activities as well as multimerization profile similar to the wild type IN, indicating that this mutation has limited impact on the conformation of the protein. Results from our HTRF assay confirmed previous studies showing that both IN W131A and IN W131D were defective for LEDGF/p75 binding in His-IN pull down assays [15,17].

We observed that the oligomerization profiles of IN mutants in solution could not completely predict their capacities to perform concerted strand transfer reaction. This is exemplified by the ability of the mutant Q168L to retain partial concerted integration activity (Figure 3B), suggesting that the monomeric IN Q168L could oligomerize in presence of DNA. We also observed that mutations of the residue Y194 strongly affected the enzymatic activity of the protein independently of their abilities to tetramerize probably by affecting directly the catalytic property of the protein (Figure 3A).

Our interaction assays indicate that IN mutants W131A and Q168L are the most deficient for TNPO3 interaction. The primarily replication defect of HIV-1 W131A takes place during reverse transcription indicating that this mutation somehow impacts the RTC conformation. Subsequently, the slight reduction in 2-LTR circles could be an indirect consequence of the reverse transcription defect. On the other hand, the HIV-1 Q168L virus displayed modest increase of 2-LTR circles level and viral replication appears to be mainly impaired at the integration step (Figures 6 and 7). Mutations of IN affecting simultaneously nuclear import and integration would have opposite effects on the accumulation of 2-LTR circles in the nucleus, resulting in a null effect. To uncoupled the phenotype of these mutant viruses on nuclear entry from the phenotype due to the defective interaction between IN and LEDGF/p75, we quantified 2-LTR circle levels in LEDGF/p75-depleted cells [71]. Similar to the results obtained in SupT1 cells, we observed a 30% decrease in the formation of 2-LTR circles for HIV-1 W131A, whereas HIV-1 Q168L accumulated near wild type amounts (Figure 7C). Therefore, the study of these two IN mutants showed that no direct correlation could be found between a defect in TNPO3 binding and the formation of 2-LTR circles. Taken together, these results suggest that the interaction between IN and TNPO3 is not directly involved at the nuclear import step, but could rather take place at a later stage in the nucleus. As quantification of 2-LTR circles is only an indirect measurement of nuclear import, these results should be confirmed using more direct methods [74,75]. Nevertheless, these results corroborate mounting evidence against a direct role of IN in controlling the shuttling of the PIC between the cytoplasm and the nucleus in dividing or non-dividing cells [76,77].

Furthermore, CA was found to be the dominant viral determinant for HIV-1 infection of non-dividing cells. The kinetic of dissociation of CA from the viral core appears to be a critical step that could control subsequent interactions with cellular karyopherins [40,41]. Recently, HIV-1 CA mutant N74D was shown to escape replication restriction observed upon TNPO3 depletion [66]. This result reinforces previous observations for a role of CA as the main viral determinant for TNPO3 dependency during HIV-1 infection [64-66]. However, whereas the importance of CA as the main determinant of TNPO3-dependency for HIV-1 replication is well supported by experimental evidence [64-66], proof of a direct interaction between CA and TNPO3 is still missing. Using TNPO3 as bait in a rebound yeast two-hybrid screening against a prey library of random HIV-1 fragments, we identified IN, but not CA, as the binding partner of TNPO3 [62,63]. However, it would be important to determine if multimerization of CA is required for TNPO3 binding. Recently, TNPO3 has been shown to pull-down CA as well as tRNA from purified virions in a RanGTP-manner, and a model has been proposed in which TNPO3 could displace CA and tRNA that remain associated to nuclear PICs in order to facilitate integration [78]. Whether this mechanism involves a direct interaction between TNPO3 and CA in the nucleus remains to be explored.

Beside the role of CA, a direct interaction between IN and TNPO3 could take place after entry of the PIC in the nucleus. Therefore, beside its role in nuclear import, TNPO3 could also be involved in nuclear events of HIV-1 replication. This model is supported by recent findings showing that depletion of TNPO3 affected a post-import step of HIV-1 replication [78], and it was recently shown that by regulating nuclear import of the PIC in a CA-dependent manner, both NUP358/RanBP2 and TNPO3 control the selection of integration sites in the chromosomes of the host cell [79]. This latter study proposed an interesting model in which nuclear translocation through the pore would result in the specific targeting of PICs in gene dense regions of the genome, a step that could required a direct interaction between IN and TNPO3.

An intriguing question remains regarding the pleiotropic effects of the mutation W131A on HIV-1 replication. Similar to our observations with HIV-1 W131A, a defect of viral cDNA synthesis was also described for the VSVg-pseudotyped HIV-1 W131A and HIV-1 W131D viruses [15,17]. IN mutants have been reported to perturb the reverse transcription step, possibly by altering the overall conformation of the RTC [80]. A direct interaction between IN and RT was reported and could account for these effects [81]. In addition, HIV-1 viruses carrying IN deletion or the C130S substitution were recently described to decrease CypA-CA interaction leading to the destabilization of the viral core [82]. Future experiments would be required to assess the kinetic of CA uncoating of IN mutated viruses depending of their mechanism of entry (*i.e*. fusion vs. endocytosis).

## Conclusion

Interactions between IN and host cellular factors control different stages of HIV replication. TNPO3 was recently described as a HIV-1 dependency factor able to bind HIV-1 IN, and RNAi studies indicated that TNPO3 participates in the import of the PIC into the nucleus [53,55,62]. Our site-directed mutagenesis analysis aimed at exploring the contribution of the interaction between IN and TNPO3 to the early stages of the viral lifecycle. The present study indicates that IN mutants W131A and Q168L previously shown to be defective for LEDGF/p75 were also impaired for TNPO3 binding, albeit to a lesser extent. However, none of these mutations significantly reduced the level of the 2-LTR circles, arguing against a role of IN-TNPO3 interaction in nuclear import.

## Methods

### Cells, viruses and infections

HeLa and 293T cells were grown in Dulbecco's modified Eagle's medium supplemented with 10% of fetal calf serum and antibiotics (100 units/ml penicillin G, 100 mg/ml streptomycin; GIBCO, Invitrogen). SupT1 and LEDGF-depleted SupT1 TL34 cells [71] were grown in RPMI 1640 supplemented with 10% of fetal calf serum and antibiotics (100 units/ml penicillin G, 100 mg/ml streptomycin; GIBCO, Invitrogen).

#### Viral production

Bru WT or IN mutant virus stocks were generated by transfecting 293T cells with pBru-derived molecular clones using PEI (Polyethylenimine, Polysciences). Cells were washed 16 h after transfection, and supernatants collected 24 h thereafter were filtered through 0.45 µm filters and aliquoted. Viral stocks were quantified by measuring the CAp24 antigen using the Innotest HIV Antigen mAb kit, accordingly to manufacturer instructions (Innogenetics).

#### Viral replication

SupT1 or Sup T1 TL34 cells were infected with viral doses corresponding to 100 ng of HIV-1 CAp24 antigen per 2.10^6 ^cells, viral replication of each mutant viruses compare to the WT was followed by monitoring the HIV-1 CAp24 in the producer cells supernatants as described above.

### Plasmids and mutagenesis

#### His_6_-tagged integrases

Mutated recombinant IN Q168L, IN W131A, IN K188Q, IN Y194E, IN Y194F were obtained by site-directed mutagenesis of the pINSD.His plasmid [83,84]. IN mutations were introduced into the pBru molecular clone by site-directed mutagenesis.

### Expression and purification of recombinant proteins

GST-LEDGF/p75 encoding sequence was introduced in the pGGWA plasmid using the gateway system (Invitrogen, Stratagene). Recombinant GST proteins were produced in *Escherichia coli *BL21. *E. coli *transformed with GST-TNPO3 or GST-LEDGF/p75 expression constructs were grown in LB medium at 37°C to an A_600 _of 0.9-1.0 prior to induction with 1 mM isopropyl-thio-D-galactopyranoside (IPTG) for 4 h at 37°C. Bacteria were harvested by centrifugation and lysed by sonication in GST lysis buffer A (1 M NaCl, 40 mM Hepes pH 7.0, 5 mM MgCl_2_, 5% glycerol, 7 mM CHAPS, 2 mM β-mercaptoethanol) supplemented with protease inhibitor mixture (Roche) and 1 mM phenylmethylsulfonyl fluoride. Lysates were first clarified by centrifugation at 18000 × *g *for 50 min at 4°C and then incubated with 500 μl glutathione-Sepharose (settled beads volume, GE Healthcare) for 1 h at 4°C. Sepharose beads were washed twice with 20 ml of cold-ice GST lysis buffer, and recombinant proteins were eluted in 500 μl of GST-lysis buffer supplemented with 20 mM reduced gluthatione pH 7.0. His_6_-tagged IN WT and IN mutant proteins were purified by nickel affinity as previously described [85]. N-terminal His_6_-tag was removed by digestion with 200 U/mg thrombin (Sigma) O/N at 4°C and injected into a 5 ml HiTrap heparin column attached to an AKTA purifier system (GE Healthcare). Bound protein was eluted with a linear gradient of 0.25 to 1.0 M NaCl, 7 mM CHAPS, 50 mM Tris HCl pH 7.4.

### His_6_-tag pull-down assay

Binding of IN to TNPO3, LEDGF/p75 or LEDGF/p75-IBD was assayed in pull-down buffer (PB): 150 mM NaCl, 25 mM Imidazole, 2 mM MgCl_2_, 0,1% NP40, 50 mM Tris HCl pH 7.4. Two μg of recombinant His_6_-tagged HIV-1 IN were incubated with 2 μg of GST, GST-TNPO3 FL, GST-LEDGF/p75 FL or GST-LEDGF/p75 IBD in 200 μl of PB. Following a 2 h incubation at 4 °C, the mixtures were centrifuged briefly at full speed to remove aggregated proteins. Samples were supplemented with 20 μl (settled beads volume) of Ni-charged resin (Bio-Rad) and with 10 μg of bovine serum albumin (BSA). The mixtures were incubated for an additional 2 h at 4°C. Beads were recovered by centrifugation and washed three times with 1 ml of ice-cold PB. Beads were boiled in 20 μl 2 × Laemmli (Sigma). Eluted proteins were separated by SDS-PAGE and detected by staining with Page blue protein staining solution (Fermentas).

### Size exclusion chromatography

Experiments were performed with 2.5 μM of untagged IN on a Superdex 200 10/300 GL column attached to an AKTA purifier system (GE Healthcare) at flow rate 0.5 ml/min in buffer containing 50 mM HEPES (pH 7.4), 750 mM NaCl, and 10% glycerol. If it was necessary, proteins were diluted in this buffer before injection. Proteins were detected by absorbance at 280 nm. The column was calibrated with the following proteins: thyroglobuline (669 kDa), apoferritine (443 kDa), β-amylase (200 kDa), ADH (150 kDa), albumin (66 kDa) and carbonic anhydrase (29 kDa) (MW-GF-100 kit, Sigma).

### HTRF^® ^assay (Homogeneous Time Resolved Fluorescence)

Assays were carried out in a black 384-halfwell microplate (Greiner) using the following Interaction Buffer (IB): 25 mM Hepes pH 7.3, 150 mM NaCl, 2 mM MgCl_2_, 0,1% BSA, 1 mM DTT. Recombinant proteins were diluted in IB and used at a final concentration of 80 nM for His-IN and 5 nM for GST-TNPO3 or GST-LEDGF/p75. Anti-GST Europium Cryptate and anti-His-XL from CisBio International were reconstituted as recommended. Antibodies were firstly diluted (1/200) in IB supplemented with 0.8 mM Potassium Fluoride, 1 mM DTT and used at a final dilution 1/400. After addition of the interacting proteins and both antibodies in a final reaction volume 20 μl, the microplate was read at different times in a PHERAstar (BMG) at 665 and 620 nm after excitation at 337 nm. The specific HTRF signal was expressed as a percentage of ΔF and calculated as follows: ΔF(%)={[(665/620) sample-(665/620) blank]/(665/620) blank}*100.

### In vitro integration assays and concerted integration

3' processing and strand transfer activity assays were carried out at 37°C, in a buffer containing 20 mM HEPES (pH 7.2), 1 mM DTT, 7.5 mM MnCl_2 _in the presence of 12.5 nM DNA substrate and 60 nM or 120 nM of IN as previously described [86]. Products were separated by electrophoresis in denaturing 18% acrylamide/urea gels. Gels were analyzed with a Molecular Dynamics STORM phosphoimager and quantified with Image Quant™ 4.1 software. Concerted integration was measured as previously described [68]. Briefly purified HIV-1 IN (2 and 5 pmoles) was pre-incubated with both the 5'-end-labeled donor DNA (15 ng) containing the pre-processed U3 and U5 LTR sequences and the target DNA plasmid pBSK^+ ^(150 ng) at 0° C for 20 min in a total volume of 5 µl. Then the reaction mixture (20 mM HEPES, pH 7.5; 10 mM DTT; 10 mM MgCl_2_; 15% DMSO; 8% PEG, 30 mM NaCl) was added and the reaction proceeded for 120 min at 37°C in a total volume of 10 µL. Incubation was stopped by adding a phenol/isoamyl alcohol/chloroform mix (24/1/25 v/v/v). The aqueous phase was loaded on a vertical 1% agarose gel in the presence of 1% bromophenol blue and 1 mM EDTA. After separation of the products, the gel was treated with 5% TCA for 20 min, dried and autoradiographed. All IN activities were quantified by scanning of the bands (half-site plus full-site integration products) after gel electrophoresis and autoradiography using the Image J software.

### Quantification of HIV-1 late reverse transcribed (LRT) viral cDNA products, two-LTR circles and integrated DNA

Prior to infection, viral stocks were treated 1 h at 37° C with 100 U per ml of DNAseI (Roche). SupT1 or SupT1 TL34 cells (6.10^6^) were infected with viral doses corresponding to 1 µg of HIV-1 CAp24 antigen per 2.10^6 ^cells in 6-wells plates with Bru WT or IN mutant viruses. At 2 h post-infection (p.i.), cells were washed twice in phosphate buffered saline (PBS) 1X, and 2.10^6 ^cells were plated in 12 wells plates in presence of Saquinavir (0.5 µM) to limit viral infection to a single round. At 7 h, 24 h and 48 h p.i. cells were harvested, washed twice in PBS 1X and DNA was extracted using the QIAamp Blood DNA Minikit (Qiagen). Quantifications of viral DNA were performed by real-time PCR using the LightCycler 480 system (Roche). The PCR reactions contained LightCycler480 Probes Master mix including the FastStart Taq DNA Polymerase, dNTPs and 6.4 mM MgCl_2_. Primers, probes, QPCR run conditions were described previously [87]. The copy number of HIV-1 LRT viral cDNA products and 2-LTR circles were determined using standard curves obtained by amplification of cloned DNA containing the matched sequences. The copy number of integrated HIV-1 DNA was determined in reference to a standard curve generated by concomitant two-stage PCR amplification of a serial dilution of the standard HeLa R7 Neo cell DNA [88]. The copy number of each viral form was normalized with the number of cells obtained by the quantification by PCR of the β-globin gene according to the manufacturer instructions (Roche). When SupT1 TL34 cells were used, LRT viral cDNA products were quantified using a Taqman probe and primers within the *env *region as described previously [72].

## Competing interests

The authors declare that they have no competing interests.

## Authors' contributions

AC, ES, OD, VP, and AS performed experiments. AC, OD, VP, and SE designed experiments. AC, OD, VP, RB, MR and SE analyzed results. AC, OD, VP, MR and SE wrote the manuscript. All authors read and approved the final manuscript.

## Supplementary Material

Additional file 1 **Figure S1. HIV-1 IN interacts with LEDGF/p75 and TNPO3 in vitro**. Recombinant His-IN was incubated with GST, GST-LEDGF/p75-IBD, GST-LEDGF/p75 and GST-TNPO3 and assayed in a Ni-NTA pull-down experiment. The pull-downed proteins were separated in SDS-PAGE gels and detected by staining with Coomassie Blue G250. The positions of protein MWM are indicated. Click here for file

Additional file 2 **Figure S2. VSVg pseudotyped HIV-1 IN mutant viruses are affected at the reverse transcription step**. HeLa cells (2.10^5^) were infected with viral doses corresponding to 1 μg of HIV-1 CAp24 antigen cells in 6-wells plates with VSVg pseudotyped Bru WT or IN mutant viruses. At 7 h p.i., cells were harvested, washed twice in PBS 1X and DNA was extracted using the QIAamp Blood DNA Minikit (Qiagen). Quantifications of LRT viral cDNA products were performed by real-time PCR using the LightCycler 480 system (Roche). Click here for file
