# Supplementary figures and images for: Mutations affecting interaction of integrase with TNPO3 do not prevent HIV-1 cDNA nuclear import
Source: Retrovirology. 2011 Dec 16;8:104. doi: 10.1186/1742-4690-8-104 (PMC3286403; doi:10.1186/1742-4690-8-104)

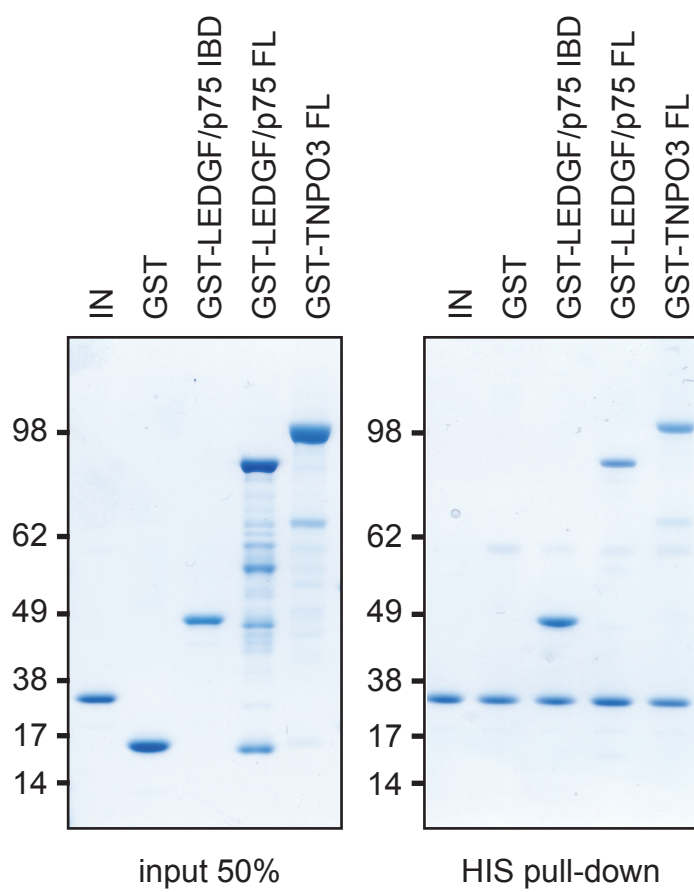

Figure S1 Cribier et al.

Supplement: Additional file 1 — Figure S1. HIV-1 IN interacts with LEDGF/p75 and TNPO3 in vitro. Recombinant His-IN was incubated with GST, GST-LEDGF/p75-IBD, GST-LEDGF/p75 and GST-TNPO3 and assayed in a Ni-NTA pull-down experiment. The pull-downed proteins were separated in SDS-PAGE gels and detected by staining with Coomassie Blue G250. The positions of protein MWM are indicated. [file 1742-4690-8-104-S1.PDF]

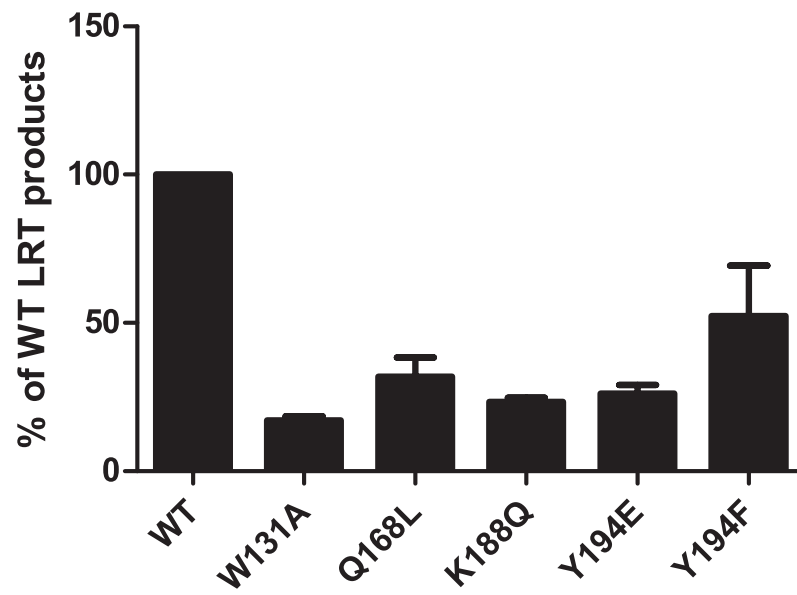

Figure S2 Cribier et al.

Supplement: Additional file 2 — Figure S2. VSVg pseudotyped HIV-1 IN mutant viruses are affected at the reverse transcription step. HeLa cells (2.105) were infected with viral doses corresponding to 1 μg of HIV-1 CAp24 antigen cells in 6-wells plates with VSVg pseudotyped Bru WT or IN mutant viruses. At 7 h p.i., cells were harvested, washed twice in PBS 1X and DNA was extracted using the QIAamp Blood DNA Minikit (Qiagen). Quantifications of LRT viral cDNA products were performed by real-time PCR using the LightCycler 480 system (Roche). [file 1742-4690-8-104-S2.PDF]
